# Long-Term Dietary *Lycium ruthenicum* Murr. Anthocyanins Intake Alleviated Oxidative Stress-Mediated Aging-Related Liver Injury and Abnormal Amino Acid Metabolism

**DOI:** 10.3390/foods11213377

**Published:** 2022-10-26

**Authors:** Shasha Chen, Honglun Wang, Na Hu

**Affiliations:** 1 Qinghai Provincial Key Laboratory of Tibetan Medicine Research and CAS Key Laboratory of Tibetan Medicine Research, Northwest Institute of Plateau Biology, Xining 810008, China; 2 School of Food and Biological Engineering, Shaanxi University of Science and Technology, Xi’an 710021, China; 3 Huzhou China-Science Innovation Centre of Plateau Biology, Huzhou 313000, China

**Keywords:** *Lycium ruthenicum* Murr., dietary anthocyanins, anti-aging function, liver-protection, serum metabolomics

## Abstract

In recent years, the relationship between *Lycium ruthenicum* Murr. anthocyanins (LRA) and health has attracted increasing attention. The purpose of this study is to investigate the anti-aging effect and mechanism of LRA through a D-galactose (DG)-induced aging rat model. Our results showed that the long-term intake of LRA, for 8 weeks, improved motor function, reduced serum aging markers, promoted the endogenous antioxidant system, and suppressed the serum inflammatory cytokines in aging rats. Besides, the LRA treatment alleviated DG-induced liver injuries by relieving the inflammation and inhibiting Fas/FasL-mediated cell death. More importantly, the abnormal serum metabolome profiles of the aging rats were restored by the LRA, relating to 38 metabolites and 44 pathways. Specifically, the LRA significantly affected the amino acid and protein-related metabolic pathways by regulating the levels of L-threonine, L-aspartic acid, glycine, L-histidine, D-homocysteine, L-homocitrulline, L-homoserine, guanidineacetic acid, and kynurenine. These results have important implications for the development of LRA as an anti-aging and liver-protective ingredient.

## 1. Introduction

The aging problem is getting much attention as the world’s aged population is increasing at a high pace. Aging is a time-dependent, multifactorial biological process that is accompanied by oxidative damage, mitochondrial dysfunction, immune-senescence, altered nutrient sensing, and so on [1]. Altogether, these alterations imperil cells and tissues, and contribute to progressive physical deterioration, functional impairments, chronic diseases, and death [2]. Oxidative stress plays a vital role in aging. The accumulation of reactive oxygen species (ROS) results in glycoxidation reactions and lipid peroxidation, which leads to the increased endogenous generation of advanced glycation and lipoxidation end products (AGEs and ALEs) [2]. Both AGEs and ALEs can induce alterations in cell signaling and function that are associated with the aging process and various age-related metabolic disorders [3]. While the aging process is inevitable and irreversible, there are numerous ways to help mitigate and delay the process. Dietary interventions are considered essential to minimize the detrimental effects of aging [4].

*Lycium ruthenicum* Murr. (LR), family Solanaceae, is an edible berry and has long been used as a functional food [5]. The representative bioactive ingredients in the fruit of LR are anthocyanins [6]. The health benefits of LR anthocyanins (LRA) have been extensively studied, such as their antioxidant [7], anti-inflammatory [8], anti-gouty arthritis [9], liver-protection [10], hypoglycemic [11], anti-obesity [12], and gut microbiota modulation [13,14] benefits. In addition, our previous work showed that LRA could relieve aging-related memory impairment and hippocampal damage in a D-galactose (DG)-induced accelerated aging rat model [15,16]. Nevertheless, additional investigations are recommended to comprehensively understand the anti-aging effects of the long-term dietary intake of LRA.

Metabolomics focuses on the identification of small molecule metabolites (MW < 1500 Da), which can be used to discover biomarkers and elucidate their metabolic mechanisms [17,18]. In fact, metabolites alterations have been linked to the aging process [19,20]. Several studies have reported changes in the serum, plasma, or blood metabolites with aging, and the metabolic pathways related to the amino acid, carbohydrate, and lipid metabolisms [21,22,23,24]. To the best of our knowledge, the metabolic function and its underlying mechanisms of LRA in the aging process have not been reported.

This study aimed to investigate the anti-aging effects of the long-term intake of LRA by analyzing the serum aging-related biomarkers and liver damage in DG-induced accelerated aging rats. The LC-MS-based metabolomics approach was used to study the effect of LRA on the serum metabolite profiles of aging rats. The underlying mechanism was explored from the perspective of anti-oxidation and anti-inflammation through the significantly affected metabolites and metabolic pathways. Our findings highlight the advantages of LR as a functional food and might provide new insights for the development of LRA as a dietary supplement or nutrient with anti-aging efficacy.

## 2. Materials and Methods

### 2.1. Chemicals and Materials

The D-galactose (DG) was purchased from Sigma-Aldrich (USA). The LR anthocyanin (LRA) sample was prepared as we described previously [15]. Briefly, the anthocyanins were obtained from dried LR fruit by ultrasound-assisted extraction using 40-fold 80% ethanol (extracted for 1 h and repeated three times). The extract was concentrated, purified with AB-8 macroporous resin, and dried by lyophilization. The LRA sample was stored at −20 °C. The purity of the anthocyanin was 87.54% of the sample, which was calculated by the pH-differential method. The anti-TNF-α antibody was obtained from Abcam Plc (Cambridge, UK) and the fluorescent anti-rabbit secondary antibody was purchased from Invitrogen Corporation (Carlsbad, USA).

### 2.2. Animals and Treatments

The DG-induced accelerated aging model has been widely applied in anti-aging therapeutic intervention studies, [25] and its practicability has been verified by our previous research [16]. Therefore, we used a DG-treated rat model to study the anti-aging effects of LRA in this study.

The Sprague–Dawley rats (female, 200 ± 20 g) were obtained from Junxing Biological Technology Co., Ltd. (Xi’an, Shaanxi, China) and housed in the animal room. After 1 week of habituation, the rats were randomly divided into three groups (*n* = 9) and treated with 0.9% saline solution (NC group), 100 mg/kg of DG (IP, DG group), and 100 mg/kg of DG (IP) plus 100 mg/kg of LRA (IG) (DG + LRA group). The dose of the LRA was chosen according to our previously published work [15]. The DG and LRA were treated once daily for 8 weeks.

All the animal assays were performed following the ARRIVE guidelines, carried out in strict accordance with the National Institutes of Health Guide for the Care and Use of Laboratory Animals (NIH Publications No. 8023, revised 1978), and approved by the committee of the Northwest Plateau Institute of Biology, CAS for animal experiments (grant number: 2020-18).

### 2.3. Evaluation of the Motor Ability of Rats

After the last treatment of DG and LRA, a swimming test was conducted to evaluate the motor ability of the rats. A round pool of 125 cm in diameter and 51 cm in height was employed, which was filled to a 30 cm depth with water (24 ± 1 °C). Each rat was allowed to swim in the pool for 90 s, and the distance (cm) was measured to calculate the swimming speed (cm/s).

### 2.4. Sample Collection

After the swimming experiment, the rats were allowed to rest for a day before being sacrificed. The rats were anesthetized by an intraperitoneal injection of 10% chloral hydrate at a dose of 0.3 mL/100 g. Blood was collected from the abdominal aorta, and the rats were immediately killed by cervical dislocation. The blood sample was left at room temperature for 1 h before being centrifuged at 3000 rpm for 5 min. The supernatant was collected and centrifuged at 4000 rpm for 10 min to separate the serum. The serum sample was frozen in liquid nitrogen and stored at −80 °C. Liver samples were harvested, of which three (*n* = 3) were stored in a 4% paraformaldehyde solution for paraffin embedding, and the remaining six were stored at −20 °C.

### 2.5. Serum Biochemical Assays

The serum levels of the advanced glycation end products (AGEs), malondialdehyde (MDA) and reduced glutathione (GSH), as well as the activities of glutathione peroxidase (GSH-Px), catalase (CAT), total superoxide dismutase (T-SOD), lactic dehydrogenase (LDH), alanine aminotransferase (ALT), and aspartate aminotransferase (AST), were detected using commercially available assay kits (Nanjing Jiancheng Bioengineering Institute, Jiangsu, China). The serum levels of metallothionein, TNF-α, IL-6, and IL-10 were detected by using the ELISA method with commercially available kits (Shanghai Enzyme-linked Biotechnology Co., Ltd., Shanghai, China).

### 2.6. Hematoxylin and Eosin (HE) Staining

The liver sample was prepared into 5 μm paraffin sections and dewaxed and rehydrated for HE staining according to the instructions of the commercial assay kit (Beyotime, Shanghai, China).

### 2.7. Immunohistochemistry

The immunohistochemistry analysis was used to measure the expression of TNF-α in the liver, as described previously [15]. The Image J program was applied to count the TNF-α positive cells in the liver.

### 2.8. qRT-PCR Analysis

The total RNA was extracted from the liver tissue (*n* = 3/per group) using the Genomic DNA Extraction Kit (Dongsheng Biotech, Guangzhou, China) and immediately reversed-transcribed into cDNA with the kit (Bio-rad, Hercules, USA). The QuantiTect SYBR^®^ Green PCR Kit (Qiagen, Hilden, Germany) was used for the quantification of the cDNA targets. The Q-PCR reaction was conducted in 96-well plates in triplicate, as we previously described [15]. The primers were as follows: TCTAGTTGGAAAGAACCGAAGG (Forward) and CCACAAACGAGATGCAATCAC (Reverse) for Fas, ATCCCTCTGGAATGGGAAGA (Forward) and CCATATCTGYCCAGTAGTGC (Reverse) for FasL, TGACAGGATACAGAAGGAGA (Forward) and TAGAGCCACCAATCCACACA (Reverse) for β-actin.

### 2.9. Extensive Targeted Metabolomics Analysis

For the LC-MS/MS analysis, 1 volume of the thawed serum sample was mixed with 3 volumes of ice-cold methanol. The mixture was whirled and centrifuged twice, and the supernatant was obtained. The UPLC-ESI-MS/MS system (UPLC, Shim-pack UFLC SHIMADZU CBM A, Shimadzu Company, Japan; MS, QTRAP^®^, SCIEX Company, USA) was employed. The analytical conditions were as follows: column temperature, 40 °C; flow rate, 0.4 mL/min; injection volume, 2 μL; solvent system, water (containing 0.04% acetic acid) and acetonitrile (containing 0.04% acetic acid); gradient program, 95:5 *v*/*v* at 0 min, 5:95 *v*/*v* at 11.0 min, 5:95 *v*/*v* at 12.0 min, 95:5 *v*/*v* at 12.1 min, and 95:5 *v*/*v* at 14.0 min. The metabolites were identified based on the standard data of Metware (Wuhan, China) and quantified according to their peak area.

### 2.10. Statistical Analysis

The GraphPad Prism 7.0 software was applied for the statistical analysis. The data were expressed as the mean ± SD. Student’s t-test and one-way ANOVA were applied to determine the significant differences. The *p* < 0.05 was considered statistically significant. 

The principal component analysis (PCA) and orthogonal partial least squares-discriminant analysis (OPLS-DA) of the metabolomics data were conducted by employing the SIMCA-P software (version 14.0). The differential metabolites were screened based on the combination of the variable importance in projection (VIP) value, *p*-value, and fold change. The Kyoto Encyclopedia of Genes and Genomes (KEGG) pathway enrichment analysis was conducted to reveal the most relevant pathways that are involved in the anti-aging effect of LRA.

## 3. Results

### 3.1. Body Weight, Motor Ability, and Serum Aging Markers

To study the effects of the long-term dietary intake of LRA during the aging process, the DG-treated rats were simultaneously administrated with LRA by gavage (once daily for 8 weeks). The rats were weighed before the dissection experiment. As displayed in Table 1, there were no significant differences in body weight among the NC, DG, and DG + LRA groups. The rats in the DG group showed impaired motor function, as seen by the significantly decreased swimming speed compared to the NC group. However, the LRA intake significantly improved the motor function of the DG-treated rats by increasing their swimming speed.

Aging is accompanied by the accumulation of oxidative products including AGEs and lipid peroxidation products (MDA) [2]. Our result showed that the DG group had significantly higher serum contents of AGEs and MDA than those in the NC group, while their levels significantly decreased after the LRA intake.

On the contrary, the serum concentrations of nonenzymatic antioxidants, including metallothionein and GSH, as well as antioxidant enzymes including GSH-Px, CAT, and T-SOD, were significantly lower in the DG group than those in the NC group. However, the concentrations of these antioxidant components were significantly increased after the LRA intake. Therefore, LRA could improve the endogenous antioxidant system of rats, thereby mitigating DG-induced oxidative stress.

Furthermore, as compared with the NC group, the serum pro-inflammatory cytokines (TNF-α and IL-6) were significantly increased, while the anti-inflammatory cytokine (IL-10) was significantly decreased in the DG group. Interestingly, the serum TNF-α, IL-6, and IL-10 were restored after the LRA intake.

### 3.2. Effect of LRA on Liver Injury

**Figure 1:**
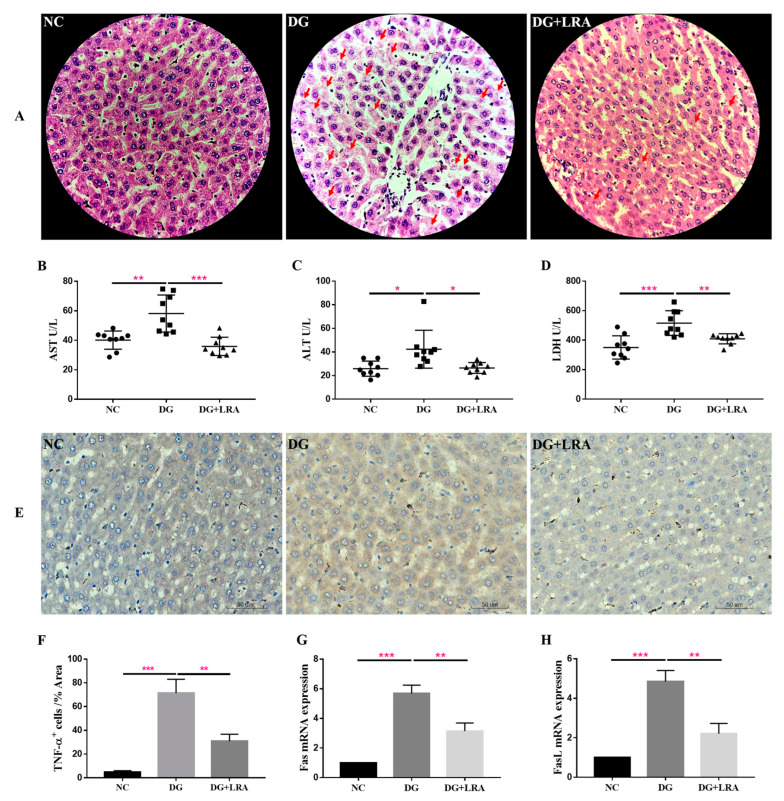
Protective effects of LRA on the liver of DG-induced aging rats. (**A**) HE staining of liver (×400). Red arrows indicate cell necrosis. (**B**–**D**) Serum levels of AST, ALT, and LDH (*n* = 9). (**E**) Immunohistochemical analysis of TNF-α expression in the liver. (**F**) Quantitative analysis for TNF-α positive cells (%Area) (*n* = 3), (**G**,**H**) mRNA expression of Fas and FasL in the liver (*n* = 3). Values are expressed as the mean ± SD. * *p* < 0.05, ** *p* < 0.01, and *** *p* < 0.001.

Since DG is mainly metabolized in the liver, it may have a great impact on the liver. The histological evaluation provided visual evidence of the effects of the DG and LRA on the liver. As presented in Figure 1A, the hepatic cells in the NC group have well-preserved cytoplasm and a prominent nucleus and nucleolus. The DG group exhibited an unclear structure of the hepatic cells and increased cell necrosis, while these histological damages were improved after the LRA treatment. This evidence implies that LRA exerts latent protective effects against DG-induced liver damage. 

AST and ALT are normally present in the hepatocytes, where they are released into the bloodstream in response to liver inflammation or dysfunction. The serum activities of the AST and ALT reflect the degree of liver injury. As shown in Figure 1B,C, the DG group has significantly higher serum activities of AST and ALT than those in the NC group. The LRA intake obviously lowered the AST and ALT serum levels in the aging rats.

LDH is an intracellular enzyme released during cell damage, and it also reflects the level of inflammation [26]. Compared with the NC group, the serum LDH concentration was significantly increased in the DG group, while the serum level of the LDH was decreased in the DG + LRA group in comparison to that in the DG group (Figure 1D). Collectively, the LRA intake exerted a protective effect against DG-induced liver injury and reduced the release of AST, ALT, and LDH.

### 3.3. Effect of LRA on DG-Induced Liver Inflammation and Fas/FasL Activation

The result of the immunohistochemical analysis revealed that the DG-induced liver inflammation, as shown by the increased TNF-α positive (TNF-α^+^) cells (Figure 1E,F). However, the expression of TNF-α in the liver was significantly down-regulated after the LRA intake, suggesting that LRA could suppress DG-induced liver inflammation.

Fas/FasL participates in cell death receptor signaling. Our qRT-PCR result showed a markedly up-regulated expression of Fas/FasL mRNA in the livers of the rats in the DG group compared with that in the NC group (Figure 1G,H). The LRA treatment down-regulated the Fas/FasL mRNA expression level in the aging rats, revealing that it could relieve DG-induced liver cell death by inhibiting the Fas/FasL activation.

### 3.4. Effects of LRA on the Serum Metabolome Profiles

**Figure 2:**
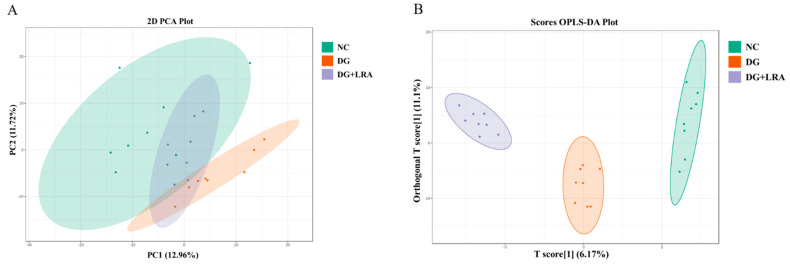

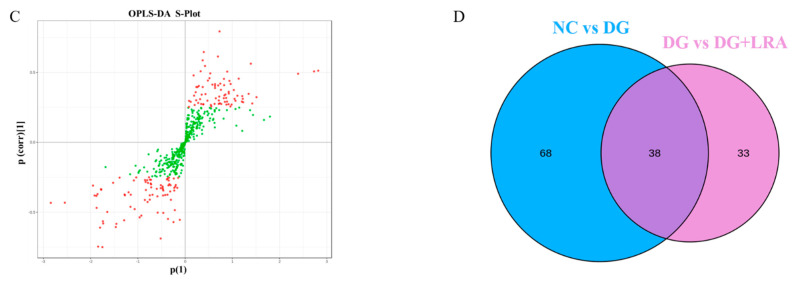
Influence of LRA on the serum metabolism of DG-induced aging rats (*n* = 8). (**A**) 2D PCA score plot. (**B**) Scores OPLS-DA plot. (**C**) Volcano plot. (**D**) Venn diagram showing the relationship between different metabolites in each group. Red dots, VIP ≥ 1; Green dots: VIP < 1.

A total of 514 metabolites were identified by an LC-MS/MS-based metabolomics analysis of the serum samples. A PCA was performed to investigate the differences in the metabolome of the NC, DG, and DG + LRA groups. The PCA score plot is shown in Figure 2A, and the scores of the first and second principal components are 12.96% and 11.72%, respectively. There was an apparent differentiation of the serum metabolomes between the NC and DG groups. Interestingly, the LRA intake recovered this alteration. To maximize the distinction among the groups, an OPLS-DA was conducted and displayed in Figure 2B,C. The established model presented a good predictive reliability (R^2^Y = 0.99, Q^2^ = 0.434).

### 3.5. Screening for the Differential Metabolites

**Figure 3:**
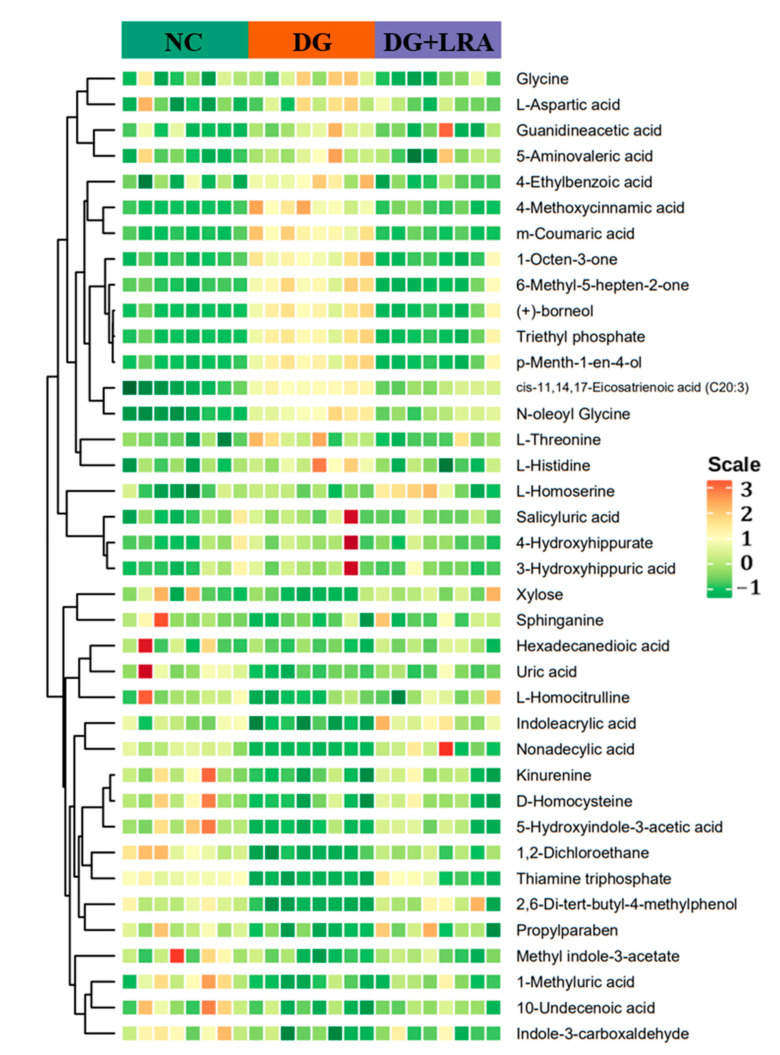
Cluster heat map of differential metabolites.

The differential metabolites were screened based on the fold change ≥ 1.2, VIP ≥ 1, and *p* < 0.05. The contents of 106 serum metabolites were significantly changed following the DG administration, among which the contents of 38 metabolites were restored after the LRA intake (Figure 2D). These 38 differential metabolites were considered potential biomarkers that are involved in the improvement of the LRA on DG-induced aging. The clustering heat map (Figure 3) illustrates the relative increase or decrease in the biomarkers in each group.

The differential metabolites were classified into different classes according to their chemical properties. As shown in Table 2, the DG up-regulated the levels of amino acid metabolomics (L-threonine, L-aspartic acid, glycine, L-histidine), organic acid metabolomics (L-homoserine, 4-methoxycinnamic acid, N-oleoyl glycine, 5-aminovaleric acid, guanidineacetic acid, 3-hydroxyhippuric acid, triethyl phosphate), lipids including *cis*-11,14,17-Eicosatrienoic acid (C20:3), benzene and substituted derivatives (m-coumaric acid, salicyluric acid, 4-hydroxyhippurate, 4-ethylbenzoic acid), and others (1-octen-3-one, 6-methyl-5-hepten-2-one, (+)-borneol, *p*-menth-1-en-4-ol), while their levels were restored to that of the NC group after the LRA intake.

Meanwhile, the levels of the amino acid metabolomics (D-homocysteine and L-homocitrulline), organic acid metabolomics (1-methyluric acid, indoleacrylic acid, kynurenine, uric acid), co-enzyme and vitamins including thiamine triphosphate, lipids (hexadecanedioic acid, nonadecylic acid, sphinganine, 10-undecenoic acid), carbohydrate including xylose, indole and its derivatives (indole-3-carboxaldehyde, 5-hydroxyindole-3-acetic acid, methyl indole-3-acetate), benzene and substituted derivatives including 2,6-di-tert-butyl-4-methylphenol, and others (propylparaben and 1,2-dichloroethane) were down-regulated by the D-galactose, and they were up-regulated by the LRA.

### 3.6. KEGG Functional Annotation and Enrichment Analysis

**Figure 4:**
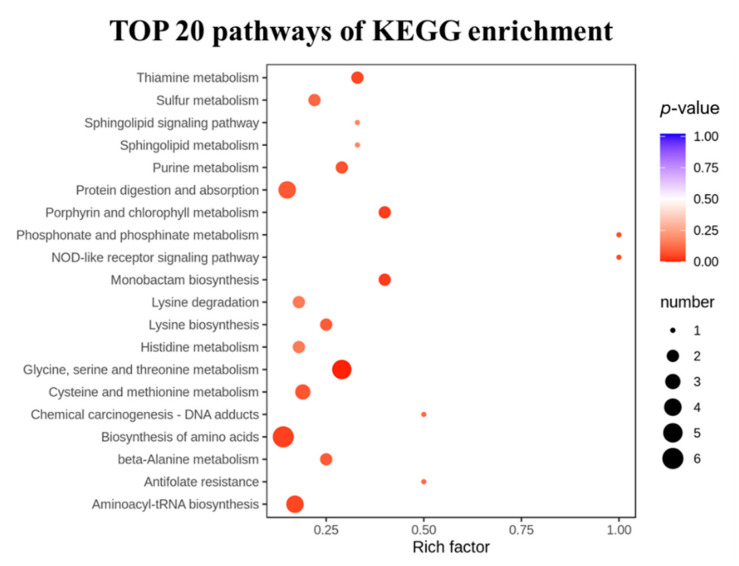
The TOP 20 significantly enriched pathways.

To analyze the effects of the LRA on the metabolic pathways of the aging rats, a metabolic pathway enrichment analysis was conducted. The selected 38 differential metabolites were annotated, based on the KEGG database (Table 2). The KEGG pathway enrichment revealed that a total of 44 pathways were associated with the anti-aging effects of the LRA. The pathways including the biosynthesis of amino acids, glycine, serine and threonine metabolism, aminoacyl-tRNA biosynthesis, ABC transporters, and cysteine and methionine metabolism were especially influenced by the LRA (Figure 4).

## 4. Discussion

Anthocyanins have health benefits including antioxidation, anti-inflammation, liver-protective effects, neuroprotective effects, and the improvement of other age-related chronic metabolic disorders [27]. This study investigated the anti-aging function of LRA in a DG-induced accelerated aging rat model. We found that long-term treatment with DG or LRA for 8 weeks had no effect on the body weight of rats. This result is different from an earlier study that reported a remarkable decrease in the body weight of mice caused by DG [28]. We consider that it may be because the dose (100 mg/kg) of DG in the current study was much lower than the dose (300 mg/kg) used in the earlier study [28]. Aging is usually accompanied by weakness and a decline in motor function. Interestingly, LRA intake could improve the impaired motor function of aging rats, as observed by the significantly faster swimming speed in the DG + LRA group than that in the DG group.

The metabolic process of DG can induce reactive oxygen species (ROS) production. Under physiological conditions, the endogenous antioxidant system (AOS), including nonenzymatic antioxidants such as metallothionein and GSH, as well as enzymatic components such as GSH-Px, SOD, and CAT, can prevent cells from being attacked by ROS. However, an impaired AOS leads to an excessive ROS accumulation, which gives rise to glycoxidation and lipid peroxidation reactions, leading to elevated AGEs and MDA levels [2]. These increased AGEs and MDA levels may cause age-related disorders, including oxidative damage in various organs, inflammation, neurodegenerative diseases, diabetes mellitus, and vascular complications [19]. Therefore, they have been suggested to be biomarkers of aging. Here, we observed increased serum levels of AGEs and MDA in the DG-induced aging rats. Conversely, the DG group had decreased serum levels of metallothionein and GSH, and reduced activities of GSH-Px, SOD, and CAT. However, the long-term intake of LRA could improve the endogenous AOS by increasing concentrations of metallothionein, GSH, GSH-Px, SOD and CAT, therefore reducing the accumulation of oxidative products in the aging rats. Sustained oxidative stress induced by DG can activate an inflammatory response via multiple signal pathways. Chronic inflammation is also a hallmark of aging, as well as a driver of many aging-related diseases [17]. Our data showed raised pro-inflammatory cytokines (TNF-α and IL-6) but depressed anti-inflammatory cytokine (IL-10) in the serum of the DG-induced aging rats. Importantly, the LRA intake could restore these serum inflammatory factors to normal levels. All these results suggested that LRA could be used as a kind of natural agent with effective antioxidant and anti-inflammatory activities.

As a crucial metabolic organ, the liver plays an important role in the detoxification of various metabolites. Liver function weakens gradually with aging, as a result of oxidative damage. As expected, our results suggested that the DG administration led to the increased liver cell death and elevated serum activities of AST, ALT, and LDH. Interestingly, the LRA exerted a protective function on the liver by mitigating these pathological changes and reducing the release of AST, ALT, and LDH. In fact, the liver-protective effects of dietary anthocyanins have been widely confirmed, such as the attenuation of diet-induced hepatic steatosis [29], an improvement of chemically-induced liver fibrosis and carcinogenesis [30], and relief of nonalcoholic fatty liver-associated inflammation [31], and the mechanisms are associated with their anti-inflammatory property.

The immunohistochemical result revealed that LRA could suppress DG-induced liver inflammation by reducing the over-expression of the proinflammatory factor (TNF-α). The impact of dietary anthocyanins on inflammation has been widely discussed [32]. Our previous work also revealed that LRA could relieve neuroinflammation [15] and gouty arthritis [9] in rats. Besides, the result of the qRT-PCR suggested that LRA could also restrain the DG-induced activation of Fas/FasL, thereby inhibiting cell death in the liver of rats. Combined with the above analysis, LRA may protect the liver from DG-induced injuries by suppressing oxidative stress and inflammation, as well as inhibiting Fas/FasL-mediated cell death. The hepatoprotective effect of LRA and its mechanism, especially in the aging process, deserve further exploration. 

Liver damage can lead to metabolic disorders, especially an amino acid metabolism disturbance in the body. The aging process is accompanied by metabolite changes [17]. Many age-related disorders, including diabetes, kidney disease, hyperlipemia, hyperuricemia, osteoporosis, and malnutrition, are connected to a metabolic dysfunction [33,34]. Our previous study showed the alteration of the hippocampus metabolome in D-galactose-induced aging rats [16]. Here, we applied an LC-MS/MS-based metabolomics approach, coupled with multivariate data analysis, to study the impact of long-term LRA intake on the serum metabolomes of DG-induced aging rats. The result suggests that the serum metabolome was notably changed in the DG group. Following the treatment with the LRA, the dysregulated serum metabolome returned to the same level as that of the NC group. This may be due to the liver-protective effect of LRA, thus reducing the serum metabolic disorders in aging rats.

Amino acids have been recognized as not only a source of energy but signal transducers that activate the signal transduction pathways. Amino acid metabolism is particularly sensitive in aging and age-associated disorders [35,36]. The serum metabolomics analysis revealed that six potential biomarkers are related to the biosynthesis of amino acids. Among them, the levels of L-threonine, L-aspartic acid, glycine, and L-histidine were up-regulated, while the levels of D-homocysteine and L-homocitrulline were down-regulated by the DG administration. Elevated levels of L-threonine, L-aspartic acid, L-histidine, and glycine are also involved in the disturbed aminoacyl-tRNA biosynthesis. Besides the abnormal levels of D-homocysteine, the L-aspartic acid, and L-homoserine indicate a disordered cysteine and methionine metabolism, which plays a role in vascular inflammation, liver disease, and chronic renal failure [37]. Additionally, an elevated L-threonine level is associated with an abnormal muscular lipid accumulation [38]. Here, the LRA intake mitigated the impacts of the DG on these metabolites and may reduce the risk of age-related hepatic, renal, cardiovascular, and cerebrovascular diseases.

We also found five potential biomarkers related to glycine, serine, and threonine metabolism. Besides the L-threonine, L-aspartic acid, and glycine that were described above, the levels of L-homoserine and guanidineacetic acid were up-regulated by the DG administration. Disrupted glycine, serine, and threonine metabolism are involved with the aging process by affecting the detoxification and redox equilibrium of the liver [39]. As expected, the LRA significantly restored these metabolites to normal levels, which proves the liver-protective effect of the anthocyanins.

The LRA also significantly recovered the levels of organic acid metabolites, such as 4-methoxycinnamic acid, N-oleoyl glycine, 5-aminovaleric acid, 3-hydroxyhippuric acid, triethyl phosphate, 1-methyluric acid, indoleacrylic acid, kynurenine, and uric acid, in the aging rats. Among them, kynurenine is a metabolite produced by the catabolism of tryptophan that regulates the host microbiota signaling immune cell responses and neuronal excitability. The decreased kynurenine may be involved in the impaired function of immune cells and elevated pro-inflammatory cytokines in the aging rats [40], while the LRA intake could restore the kynurenine level in the aging rats. The gut microbiota are an important source of kynurenine, thus, LRA may restore the kynurenine level by the regulation of intestinal microbiota in aging rats [13,14]. The anti-aging mechanism of LRA through the modification of the gut microbiota needs further study.

As discussed above, LRA exerted health promotion effects in DG-induced aging rats mainly through modulating the amino acid and protein-related metabolic pathways in response to aging-related disorders. Another functional resource, astaxanthin, has also been found to correct problems in amino acid metabolism during aging by regulating levels of N-acetyl-L-leucine, N-acetyl-L-tyrosine, and methionine [35]. Therefore, it is of great significance to study the mechanisms of functional foods with anti-aging functions by regulating amino acid metabolism.

## 5. Conclusions

In the current study, the anti-aging functions of LRA were evaluated in a DG-induced accelerated aging rat model. The LRA intake significantly reduced the serum levels of the aging-related markers, enhanced the endogenous antioxidant system (AOS), and inhibited inflammation in the aging rats. Moreover, the LRA could reduce DG-induced liver damage by suppressing pro-inflammatory factors and inhibiting Fas/FasL-mediated cell death. In addition, the LRA could restore the abnormal serum metabolome profiles of aging rats. Specifically, the LRA significantly affected the amino acid and protein-related metabolic pathways by regulating the levels of L-threonine, L-aspartic acid, glycine, L-histidine, D-homocysteine, L-homocitrulline, L-homoserine, guanidineacetic acid, and kynurenine. In conclusion, LRA probably resists oxidative stress and inflammation, relieves liver damage, and improves abnormal amino acid metabolisms, therefore reducing aging-related disorders.

## Figures and Tables

**Table 1:** Body weight, swimming speed, and serum aging-related biomarkers including AGEs, MDA, metallothionein, GSH, antioxidant enzymes, and inflammatory factors.

Group	NC	DG	DG + LRA
Body weight (g)	309.4 ± 12.8	316.6 ± 11.0	313.6 ± 13.8
Swimming speed (cm/s)	31.80 ± 2.36	28.61 ± 3.02 ^#^	32.63 ± 1.95 **
AGEs (pg/mL)	267.12 ± 35.44	305.52 ± 28.27 ^#^	250.18 ± 33.44 **
MDA (nmol/mL)	2.48 ± 0.34	3.33 ± 0.28 ^###^	2.56 ± 0.28 ***
Metallothionein (ng/mL)	75.09 ± 19.77	29.06 ± 6.57 ^##^	48.68 ± 11.28 *
GSH (nmol/mL)	436.24 ± 66.61	184.50 ± 44.08 ^###^	337.88 ± 51.73 ***
GSH-Px (U/mL)	31.56 ± 5.05	20.02 ± 3.75 ^###^	28.76 ± 4.86 ***
CAT (U/mL)	8.00 ± 1.07	3.86 ± 1.31 ^###^	7.62 ± 1.56 ***
T-SOD (U/mL)	64.02 ± 4.13	51.47 ± 5.75 ^###^	58.90 ± 7.71 *
TNF-α (pg/mL)	126.30 ± 13.19	231.70 ± 31.29 ^###^	138.09 ± 17.44 ***
IL-6 (pg/mL)	35.32 ± 7.70	58.46 ± 8.67 ^##^	39.71 ± 8.97 *
IL-10 (pg/mL)	36.98 ± 6.81	12.61 ± 5.32 ^###^	31.78 ± 14.40 **

NC, normal control; DG, D-galactose; LRA, *Lycium ruthenicum* Murr. anthocyanins. Values are expressed as the mean ± SD (*n* = 9). # Significantly different from NC group. * Significantly different from DG group. ^#^ or *, *p* < 0.05, ** or ^##^, *p* < 0.01, *** or ^###^, *p* < 0.001.

**Table 2:** The serum differential metabolites and their KEGG enrich pathways.

Class	Metabolites	Change ^#^	Chang *	KEGG PATHWAYS
Amino acid	L-Threonine	up	down	ko00260, ko00261, ko00290, ko00860, ko00970, ko01100, ko01230, ko02010, ko04974, ko04978
	L-Aspartic acid	up	down	ko00220, ko00250, ko00260, ko00261, ko00270, ko00300, ko00340, ko00410, ko00760, ko00770, ko00970, ko01100, ko01200, ko01210, ko01230, ko02010, ko04080, ko04974, ko05230
	Glycine	up	down	ko00120, ko00230, ko00260, ko00310, ko00440, ko00480, ko00630, ko00730, ko00860, ko00970, ko01100, ko01200, ko01230, ko02010, ko04080, ko04721, ko04974, ko04978, ko05230
	L-Histidine	up	down	ko00340, ko00410, ko00970, ko01100, ko01230, ko02010, ko04974, ko05230
	D-Homocysteine	down	up	ko00270, ko00920, ko01100, ko01230, ko01523, ko04621
	L-Homocitrulline	down	up	-
Organic acid	L-Homoserine	up	down	ko00260, ko00270, ko00300, ko00920, ko01100, ko01230
	4-Methoxycinnamic acid	up	down	-
	N-oleoyl Glycine	up	down	-
	5-Aminovaleric acid	up	down	ko00310, ko00330, ko01100
	Guanidineacetic acid	up	down	ko00260, ko00330, ko01100
	3-Hydroxyhippuric acid	up	down	-
	Triethyl phosphate	up	down	-
	1-Methyluric acid	down	up	ko00232
	Indoleacrylic acid	down	up	-
	Kinurenine	down	up	ko00380, ko01100, ko05143
	Uric acid	down	up	ko00230, ko01100, ko04976
Co-Enzyme & vitamin	Thiamine triphosphate	down	up	ko00730, ko01100
Lipid	*cis*-11,14,17-Eicosatrienoic acid (C20:3)	up	down	ko01040
	Hexadecanedioic acid	down	up	ko01100
	Nonadecylic acid	down	up	-
	Sphinganine	down	up	ko00600, ko01100, ko04071
	10-Undecenoic acid	down	up	-
Carbohydrate	Xylose	down	up	-
Indole and its derivatives	Indole-3-carboxaldehyde	down	up	-
	5-Hydroxyindole-3-acetic acid	down	up	ko00380, ko01100, ko04726
	Methyl indole-3-acetate	down	up	-
Benzene and substituted derivatives	m-Coumaric acid	up	down	ko00360, ko01100
	Salicyluric acid	up	down	-
	4-Hydroxyhippurate	up	down	-
	4-Ethylbenzoic acid	up	down	-
	2,6-Di-tert-butyl-4-methylphenol	down	up	-
Others	1-Octen-3-one	up	down	-
	6-Methyl-5-hepten-2-one	up	down	-
	(+)-borneol	up	down	-
	*p*-Menth-1-en-4-ol	up	down	-
	Propylparaben	down	up	-
	1,2-Dichloroethane	down	up	ko01100, ko05204

^#^, change trend of DG vs. NC. *, change trend of DG + LRA vs. DG.

## Data Availability

All data are reported in this manuscript.
